# Analysis of the relationship between two-way social support and health based on a latent profile analysis among migrant older adults in China: a cross-sectional study

**DOI:** 10.3389/fpubh.2026.1885124

**Published:** 2026-09-09

**Authors:** Meijuan Cao, Shumin Wang, Yaling Zeng, Riyu Huang, Xiaojuan Xu, Yiyun Ma

**Affiliations:** School of Medicine and Nursing, Huzhou Normal University, Huzhou, Zhejiang, China

**Keywords:** cross-sectional study, health, latent profile analysis, migrant older adults, two-way social support

## Abstract

**Background:**

Two-way social support, involving both receiving and providing support, is an important psychosocial factor influencing older adults’health. Migrant older adults may face increased health risks due to migration-related disruption of social networks, making social support particularly important. However, existing studies have mainly focused on received support, with limited attention to patterns of both support receipt and provision. This cross-sectional study aimed to identify latent classes of two-way social support and examine their associations with health among migrant older adults.

**Methods:**

A cross-sectional study design was employed, and a total of 359 migrant older adults were recruited from several communities in Huzhou, China, using convenience sampling. All participants completed a general information questionnaire, the Brief 2-Way Social Support Scale, and a health survey questionnaire. Latent profile analysis (LPA) was performed to identify subgroups of two-way social support among migrant older adults, and multivariate logistic regression analysis was used to examine the associations between the identified latent classes and health outcomes.

**Results:**

Four latent classes of two-way social support were identified using LPA: the *lack of two-way social support* class (15.6%), the *mild support-providing tendency* class (25.6%), the *support-providing dominant* class (44.8%), and the *balanced two-way social support* class (14.0%). Differences in self-rated health across latent classes of two-way social support were statistically significant (*χ*^2^ = 72.487, *p* < 0.001): the balanced two-way social support group (C4) had the highest proportion of self-rated good health, the mild support-providing tendency group (C2) had the highest proportion of fair health, and the lack of two-way social support group (C1) had the highest proportion of poor health.

**Conclusion:**

The findings indicate that two-way social support among migrant older adults can be classified into four distinct patterns. Those lacking two-way support face the greatest health risk, whereas those with balanced support show the best health status. Latent profile analysis may help identify high-risk groups and guide stratified interventions to promote equitable and sustainable healthy ageing.

## 1 Introduction

With the accelerating global population ageing and increasing mobility, the number of migrant older adults has significantly increased in recent years (1, 2). According to the 2020 Seventh National Population Census of China, the number of migrant older adults was 33.27 million, accounting for approximately 8.85% of the total migrant population and representing a 2.6-fold increase compared with a decade earlier (3). Migrant older adults are typically defined as individuals who relocate from their place of origin to other cities to accompany their adult children, pursue employment, or meet ageing-related needs (4). In this context, migrant older adults encounter multiple challenges stemming from the interaction between migration-related social network disruption and the ageing process, resulting in increased difficulties in social integration, access to health care services, and psychological adaptation; consequently, they are especially vulnerable to adverse health outcomes, and their health status has garnered increasing attention.

Social support, as a significant psychosocial determinant in the lives of older adults, has been extensively studied and confirmed to be closely associated with physical health, mental health, and quality of life (5–7). For migrant older adults, the migration process often weakens pre-existing social support networks, increasing their reliance on emotional, instrumental, and informational support from family members, communities, and institutional sources in destination areas to cope with health risks and daily life stressors (8). Moreover, migrant older adults also provide support to others through household labour, intergenerational caregiving, neighbourhood mutual assistance, and volunteer activities, thereby reshaping their social roles within newly formed social networks (9). Receiving support may facilitate access to health care, promote health-related behaviours, and alleviate psychological stress, while providing support may enhance self-efficacy, social participation, and a sense of meaning in life, thus positively affecting both physical and mental health (10, 11). The concept of active ageing proposed by the World Health Organization further underscores the synergistic roles of health and social participation later in life (12). In contrast to traditional perspectives, which conceptualize social support as a unidirectional resource received by individuals, two-waysocial support captures the interactive processes in which the receipt of support co-exists with the provision of support. Its reciprocal nature has been shown to be associated with older adults’ health status and quality of life (10, 13). For migrant older adults, different forms of two-way social support may play distinct roles in health adaptation and social integration, making it particularly important to identify which support patterns are most conducive to health.

Although existing studies generally acknowledge the importance of social support in promoting older adults’ health, research on two-way social support among migrant older adults remains relatively limited. On the one hand, most studies focusing on migrant older adults have emphasized the receipt of support, with far less attention paid to the provision of support (14–17). On the other hand, empirical research on two-way social support has largely been conducted among relatively residentially stable older populations, leaving uncertainty as to whether these findings are applicable to older adults who experience migration and social network restructuring (10, 18). Previous research has primarily utilized variable-centred analytical strategies, which may be inadequate for uncovering the heterogeneity in health outcomes associated with different configurations of social support.

To address these gaps and identify latent heterogeneity in the social support structures of migrant older adults, this study adopts latent profile analysis (LPA), a person-centred exploratory statistical approach capable of identifying distinct configurations of two-way social support within complex data and examining their differential associations with health outcomes (19, 20). This approach overcomes the constraints of prior research based on single-dimensional measures and facilitates a more nuanced examination of two-waysocial support patterns and their health implications among migrant older adults. Building on this approach, the present study aims to delineate the typological characteristics of two-way social support, explore the health implications of different latent support profiles, and propose targeted strategies to enhance social integration and promote healthy ageing, thereby improving overall quality of life. Moreover, the findings of this study are expected to provide empirical support to guide community health workers in developing individualized and precise health promotion interventions tailored to different two-way social support profiles among migrant older adults.

## 2 Methods

### 2.1 Study design and participants

Ethical approval for this study was obtained from the Ethics Committee of the School of Medicine and Nursing, Huzhou Normal University (Approval No. 202401–04). A cross-sectional design was adopted, and migrant older adults were recruited from multiple communities in Huzhou City, Zhejiang Province, China, using convenience sampling. Located in the Yangtze River Delta region, Huzhou is characterized by high population mobility and a high level of population ageing, and its migrant older adult population includes both intra-provincial and inter-provincial migrants, representing a typical profile of migrant older adults in contemporary China. Based on the retirement-age criteria adopted during the study period in China, the age thresholds were set at ≥60 years for males and ≥55 years for females (21). The inclusion criteria were as follows: (1) age ≥55 years for females or ≥60 years for males; (2) having resided in Huzhou for more than 6 months; (3) having normal language comprehension and clear consciousness; and (4) providing written informed consent and participating in the study on a voluntary basis. The exclusion criteria were as follows: (1) diagnosed with mental illness; (2) suffering from critical or severe physical illness; (3) having severe limitations in activities of daily living; or (4) having serious communication disorders. According to Kendall’s sample size estimation method, a total of 31 predictor variables were included in the study, and the required sample size was estimated to be 5–10 times the number of variables (22). Considering a 15% invalid response rate, the final target sample size was adjusted to 169–357 participants. From the 384 questionnaires distributed, 359 valid responses were obtained, representing an effective response rate of 93.5%.

### 2.2 Measures

#### 2.2.1 Two-way social support

The Brief 2-Way Social Support Scale (Brief 2-Way SSS) was originally developed by Obst in 2019 and later translated and culturally adapted into Chinese by Cui et al. (23). This instrument assesses two-way social support across four domains: received and provided emotional support and received and provided instrumental support. The scale contains 12 items, each rated on a 5-point Likert scale (1 = strongly disagree to 5 = strongly agree). Total scores range from 12 to 60, with higher values indicating stronger levels of mutual social support. In this study, the Cronbach’s α coefficient for the scale was 0.929.

#### 2.2.2 Health

Health status among migrant older adults was assessed using two self-reported indicators: (1) self-rated health, determined using the item “How would you rate your overall health?”; and (2) chronic disease status, measured by the question “Have you been diagnosed with any chronic disease by a physician?”.

#### 2.2.3 Covariates

Based on the framework of the Convoy Model of Social Relations (24) and the literature (25–27), a total of 15 factors related to sociodemographic, socioeconomic, behavioral, and migration-related characteristics that influence the subgroups of two-way social support in migrant older adults were identified, including: gender, age, educational level, marital status, household registration, religion, living arrangement, monthly personal income, medical expense payment, physical exercise, number of medications, duration of migration, scope of migration, reason for migration, and intention to stay. All the factors were involved in the General information questionnaire.

### 2.3 Quality control

Eligible participants were identified on the basis of the established inclusion and exclusion criteria. During the survey, trained investigators explained the purpose, significance, and instructions of the study to the migrant older adults in a face-to-face manner. On a voluntary basis, participants completed the questionnaires anonymously and independently. Considering the possible differences in educational level and cognitive ability among migrant older adults, data were collected through face-to-face interviews to ensure comprehension and accuracy. The completed questionnaires were collected on-site, and the investigators carefully checked each one to ensure completeness and data quality.

### 2.4 Statistical analysis

SPSS version 27.0 was used to perform descriptive statistical analyses and examine bivariate correlations. Latent profile analysis (LPA) was conducted using Mplus 8.0 to identify potential subgroups of two-way social support. Models with one to five latent classes were successively fitted and compared. Multiple fit indices, including the Akaike information criterion (AIC), Bayesian information criterion (BIC), and adjusted Bayesian information criterion (aBIC), were used to evaluate model fit, where smaller values suggest improved fit. Classification accuracy was assessed using entropy, with values closer to 1 indicating more distinct class separation (28). The Lo–Mendell–Rubin adjusted likelihood ratio test (LMRT) and bootstrap likelihood ratio test (BLRT) were applied to examine the improvement of the k-class model over the (k–1)-class model, with *p* < 0.05 suggesting statistically significant improvement (29). In addition, the posterior probabilities of class membership were examined to further validate the classification accuracy; a mean posterior probability ≥ 0.80 was considered indicative of good classification reliability (30). Once the optimal number of classes was determined, each class was labelled according to its most salient characteristics to ensure distinct conceptual interpretation. Subsequently, the health status of migrant older adults across the four latent classes was described using frequencies and percentages. Chi-square analyses were conducted to assess variations in health distribution across different latent classes of two-way social support. Finally, multivariate logistic regression analysis was applied, with self-rated health as the outcome variable and latent classes of two-way social support entered as independent variables. Statistical significance was determined at a two-tailed *p* value of < 0.05.

## 3 Results

### 3.1 Classification and naming of LPA-based two-way social support in migrant older adults

LPA was conducted on the two-way social support of 359 migrant older adults. Latent class models ranging from one to five classes were fitted sequentially; the fit indices are summarized in Table 1. As the number of latent classes increased from C1 to C5, the AIC, BIC, and aBIC values continuously decreased (e.g., the AIC decreased from 7865.984 to 4993.761), indicating improved model fit; and (2) the BLRT remained significant across all the model comparisons (*p* < 0.001). When four classes were retained, the LMR test results became nonsignificant (*p* > 0.05), suggesting that the four-class model did not statistically outperform the three-class model. However, the four-class model had the highest entropy value, implying better classification accuracy than both the three- and five-class models. The four-class (C1–C4) solution was selected after considering statistical indicators, model simplicity, and interpretability. The average posterior classification probabilities ranged from 95.6 to 99.0%, indicating good model reliability (see Table 2).

**Table 1:** Fit indices for five models using latent profile analysis (*n* = 359).

model	AIC	BIC	aBIC	Entropy	LMR	BLRT	Class Probability
1C	7865.984	7959.183	7883.043	–	–	–	1
2C	6430.538	6574.221	6456.839	0.907	<0.001	<0.001	0.460/0.540
3C	6067.842	6262.008	6103.383	0.920	0.020	<0.001	0.226/0.329/0.445
4C	5844.258	6088.907	5889.040	0.957	0.201	<0.001	0.156/0.256/0.448/0.140
5C	4993.761	5288.894	5047.784	0.938	<0.001	<0.001	0.228/0.100/0.231/0.167/0.273

**Table 2:** Probability of correct classification for each category (*n* = 359).

Profile	C1	C2	C3	C4
C1	0.965	<0.001	<0.001	0.035
C2	<0.001	0.956	0.044	<0.001
C3	<0.001	0.010	0.990	<0.001
C4	0.021	<0.001	<0.001	0.979

**Figure 1:**
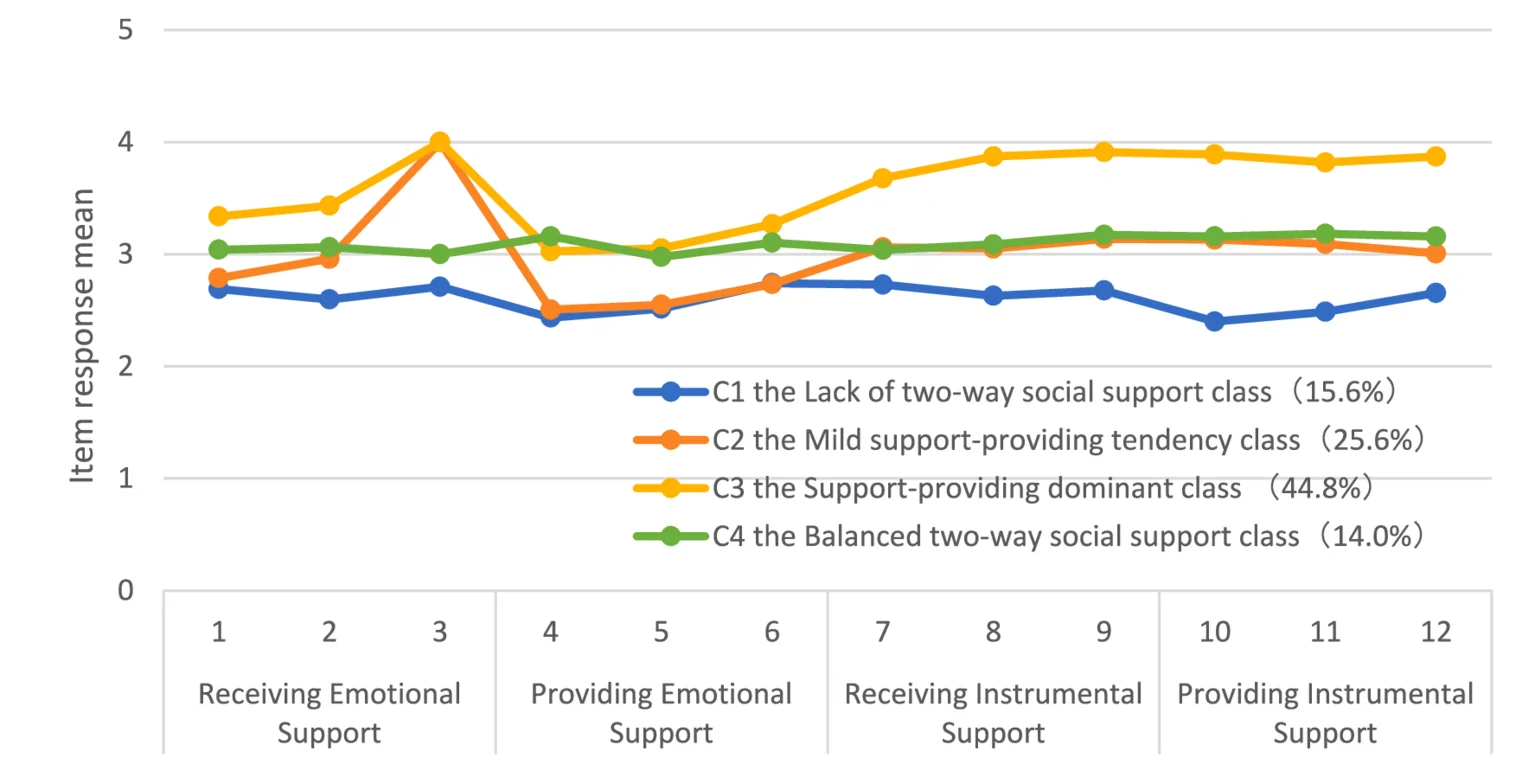
Mean Two-Way Social Support Scale score within each item.

Based on the LPA results, the mean scores of the four classes across the 12 items of two-way social support are presented in Figure 1. Items 1–3 represent received emotional support, items 4–6 represent provided emotional support, items 7–9 represent receiving instrumental support, and items 10–12 represent providing instrumental support. The identified profiles were classified and named according to their relative score patterns across the receiving and providing dimensions of emotional and instrumental support. Participants in C1 scored low on both providing and receiving support across all four dimensions and were classified as the dual-deficiency type, accounting for 15.6% (*n* = 56). Participants in C2 showed slightly higher scores in giving than in receiving support; however, the difference was relatively small and were classified as the mildly support-providing type, accounting for 25.6% (*n* = 92). Participants in C3 exhibited markedly higher scores for providing support than for receiving support, particularly in providing instrumental support, this pattern indicates a clear imbalance between support provision and receipt, with migrant older adults in this class acting primarily as support providers within their social networks. Therefore, this profile was labeled the support-providing dominant type. Notably, C3 accounted for the largest proportion of the sample (44.8%, *n* = 161), suggesting that this asymmetric pattern of two-way social support was relatively common among the participants. Participants in C4 had balanced scores (around 3 across all dimensions) in both the giving and receiving dimensions and were classified as the balanced social support type, accounting for 14.0% (*n* = 50).

### 3.2 Participants characteristics of migrant older adults across different latent classes

Table 3 presents the participants’demographic characteristics. In this study, females slightly outnumbered males, and most participants were between 60 and 69 years old (60.4%), with the majority having an education level of primary school or below (45.1%). In terms of marital status, most participants in all the classes except the lack of two-way social support class were married. Regarding household registration, the lack of two-way social support class was predominantly from rural areas (98.2%), whereas the support-providing dominant class had a greater proportion of urban residents (59.6%). Economically, a large proportion of participants reported earning between 1,001 and 3,000 RMB per month (50.1%); however, those in the lack of two-way social support class were more likely to pay medical expenses out of pocket (92.9%), while those in the dominant class relied more on medical insurance (73.3%). In terms of living arrangements, individuals in the lack of two-way social support class tended to live alone (87.5%), whereas those in the balanced social support class were more likely to live with their children (88.0%). Regarding exercise and medication use, participants in the lack of two-way social support class exercised less frequently (87.5%) and had higher rates of polypharmacy, whereas those in the support-providing dominant class showed a higher proportion of regular exercise. Regarding migration characteristics, the lack of two-way social support class primarily consisted of short-term intra-county migrants (100.0%); the support-providing dominant class included mainly intercity migrants (55.3%) who often provided childcare for grandchildren (67.7%), while the balanced class had migrated mainly for retirement purposes (90.0%).

**Table 3:** Differences in demographic characteristics among migrant older adults across latent classes.

Variables	Whole sample (*n* = 359)	C1 (*n* = 56)	C2 (*n* = 92)	C3 (*n* = 161)	C4 (*n* = 50)	*χ* ^2^	*p*
Gender						2.469	0.481
Male	164 (45.7%)	21 (37.5%)	459 (48.9%)	77 (47.8%)	21 (42.0%)		
Female	195 (54.3%)	35 (62.5%)	47 (51.1%)	84 (52.2%)	29 (58.0%)		
Age (year)						18.51	0.03
55 ~ 59	40 (11.1%)	5 (8.9%)	11 (12.0%)	20 (12.4%)	4 (8.0%)		
60 ~ 69	217 (60.4%)	24 (42.9%)	62 (67.4%)	98 (60.9%)	33 (66.0%)		
70 ~ 79	92 (25.6%)	24 (42.9%)	15 (16.3%)	40 (24.8%)	13 (26.0%)		
≥80	10 (2.8%)	3 (5.4%)	4 (4.3%)	3 (1.9%)	0 (0.0%)		
Education						14.889	0.094
Primary school or below	162 (45.1%)	37 (66.1%)	40 (43.5%)	69 (42.9%)	16 (32.0%)		
Junior high school	122 (34.0%)	13 (23.2%)	33 (35.9%)	54 (33.5%)	22 (44.0%)		
Senior high school (including vocational secondary school)	45 (12.5%)	3 (5.4%)	12 (13.0%)	23 (14.3%)	7 (14.0%)		
College degree or above	30 (8.4%)	3 (5.4%)	7 (7.6%)	15 (9.3%)	5 (10.0%)		
Marital status						24.316	<0.001
With spouse	299 (83.3%)	34 (60.7%)	80 (87.0%)	141 (87.6%)	44 (88.0%)		
Without spouse	60 (16.7%)	22 (39.3%)	12 (13.0%)	20 (12.4%)	6 (12.0%)		
Household registration						86.341	<0.001
Urban	126 (35.1%)	1 (1.8%)	14 (15.2%)	96 (59.6%)	15 (30.0%)		
Rural	233 (64.9%)	55 (98.2%)	78 (84.8%)	65 (40.4%)	35 (70.0%)		
Monthly personal income (CNY)						27.837	<0.001
≤1,000	18 (5.0%)	7 (12.5%)	6 (6.5%)	4 (2.5%)	1 (2.0%)		
1,001 ~ 3,000	180 (50.1%)	35 (62.5%)	42 (45.7%)	86 (53.4%)	17 (34.0%)		
3,001 ~ 5,000	85 (23.7%)	10 (17.9%)	21 (22.8%)	34 (21.1%)	20 (40.0%)		
≥5,001	76 (21.2%)	4 (7.1%)	23 (25.0%)	37 (23.0%)	12 (24.0%)		
Medical expense payment						86.605	<0.001
Medical insurance	192 (53.5%)	4 (7.1%)	39 (42.4%)	118 (73.3%)	31 (62.0%)		
Out-of-pocket	161 (44.8%)	52 (92.9%)	52 (56.5%)	39 (24.2%)	18 (36.0%)		
Other	6 (1.7%)	0 (0.0%)	1 (1.1%)	4 (2.5%)	1 (2.0%)		
Religion						117.378	<0.001
Yes	101 (28.1%)	0 (0.0%)	7 (7.6%)	91 (56.5%)	3 (6.0%)		
No	258 (71.9%)	56 (100.0%)	85 (92.4%)	70 (43.5%)	47 (94.0%)		
Living arrangement						264.413	<0.001
Living with spouse and children	29 (8.1%)	0 (0.0%)	9 (9.8%)	20 (12.4%)	0 (0.0%)		
Living with spouse	131 (36.5%)	0 (0.0%)	29 (31.5%)	97 (60.2%)	5 (10.0%)		
Living with children	120 (33.4%)	7 (12.5%)	36 (39.1%)	33 (20.5%)	44 (88.0%)		
Living alone	79 (22.0%)	49 (87.5%)	18 (19.6%)	11 (6.8%)	1 (2.0%)		
Physical exercise						343.541	<0.001
Regular exercise	106 (29.5%)	0 s(0.0%)	29 (31.5%)	77 (47.8%)	0 (0.0%)		
Occasional exercise	84 (23.4%)	0 (0.0%)	22 (23.9%)	57 (35.4%)	5 (10.0%)		
Previously exercised but not currently	107 (29.8%)	7 (12.5%)	32 (34.8%)	24 (14.9%)	44 (88.0%)		
No exercise	62 (17.3%)	49 (87.5%)	9 (9.8%)	3 (1.9%)	1 (2.0%)		
Number of medications						310.114	<0.001
None	82 (22.8%)	0 (0.0%)	15 (16.3%)	67 (41.6%)	0 (0.0%)		
One	79 (22.0%)	0 (0.0%)	20 (21.7%)	52 (32.3%)	7 (14.0%)		
Two	135 (37.6%)	7 (12.5%)	46 (50.0%)	40 (24.8%)	42 (84.0%)		
≥Three	63 (17.5%)	49 (87.5%)	11 (12.0%)	2 (1.2%)	1 (2.0%)		
Duration of migration						320.389	<0.001
0 ~ 1 year	57 (15.9%)	41 (73.2%)	7 (7.6%)	8 (5.0%)	1 (2.0%)		
1 ~ 3 year	125 (34.8%)	7 (12.5%)	45 (48.9%)	29 (18.0%)	44 (88.0%)		
3 ~ 5 year	38 (10.6%)	0 (0.0%)	9 (9.8%)	27 (16.8%)	2 (4.0%)		
5 ~ 10 year	131 (36.5%)	0 (0.0%)	31 (33.7%)	97 (60.2%)	3 (6.0%)		
>10 year	8 (2.2%)	8 (14.3%)	0 (0.0%)	0 (0.0%)	0 (0.0%)		
Scope of migration						182.909	<0.001
Inter-provincial	72 (20.1%)	0 (0.0%)	8 (8.7%)	61 (37.9%)	3 (6.0%)		
Inter-city	139 (38.7%)	0 (0.0%)	32 (34.8%)	89 (55.3%)	18 (36.0%)		
Inter-district/county	148 (41.2%)	56 (100.0%)	52 (56.5%)	11 (6.8%)	29 (58.0%)		
Reason for migration						320.878	<0.001
Caring for grandchildren	126 (35.1%)	0 (0.0%)	13 (14.1%)	109 (67.7%)	4 (8.0%)		
Employment/agricultural work	13 (3.6%)	0 (0.0%)	8 (8.7%)	5 (3.1%)	0 (0.0%)		
Older adult care	153 (42.6%)	8 (14.3%)	55 (59.8%)	45 (28.0%)	45 (90.0%)		
Other	67 (18.7%)	48 (85.7%)	16 (17.4%)	2 (1.2%)	1 (2.0%)		
Intention to stay						58.077	<0.001
Willing	90 (25.1%)	4 (7.1%)	24 (26.1%)	59 (36.6%)	3 (6.0%)		
Uncertain	162 (45.1%)	28 (50.0%)	45 (48.9%)	74 (46.0%)	15 (30.0%)		
Unwilling	107 (29.8%)	24 (42.9%)	23 (25.0%)	28 (17.4%)	32 (64.0%)		

In addition, Table 3 shows that no significant differences were observed in the distributions of gender and educational level (*p* = 0.481 and *p* = 0.094, respectively), whereas the four latent profiles of two-way social support differed significantly in terms of age, marital status, household registration type, monthly personal income, medical expense payment method, religion, living arrangement, physical exercise, number of medications, duration of migration, scope of migration, reason for migration, and intention to stay (all *p* < 0.05).

### 3.3 Association of two-way social support with health among migrant older adults

The chi-square test results revealed that the distribution of self-rated health among migrant older adults with different latent classes of two-way social support was statistically significant (*χ*^2^ = 72.487, *p* < 0.001), whereas the distribution of chronic disease status was not statistically significant (*χ*^2^ = 8.264, *p* = 0.219), as shown in Table 4.

**Table 4:** Differences in health among older adults by latent class.

Variables	C1	C2	C3	C4	*χ* ^2^	*p*
Self-rated health					72.487	<0.001
Healthy	11 (19.6%)	30 (32.6%)	91 (56.5%)	41 (82.0%)		
Fair health	22 (39.3%)	50 (54.3%)	49 (30.4%)	6 (12.0%)		
Unhealthy	23 (41.1%)	12 (13.1%)	21 (13.1%)	3 (6.0%)		
Disease status					8.264	0.219
With chronic disease(s)	32 (57.1%)	40 (43.5%)	71 (44.1%)	21 (42.0%)		
Without chronic disease(s)	23 (41.1%)	52 (56.5%)	85 (52.8%)	29 (58.0%)		
No medical consultation	1 (1.8%)	0 (0.0%)	5 (3.1%)	0 (0.0%)		

Continuous variables that were statistically significant in the univariate analysis were standardized. Before constructing the multivariable logistic regression model, collinearity among the independent variables was assessed using tolerance values and variance inflation factors (VIFs). As shown in Table 5, the tolerance values ranged from 0.355 to 0.932, while the VIF values ranged from 1.073 to 2.813. All variables met the criteria of tolerance > 0.1 and VIF < 10, indicating no substantial multicollinearity among the independent variables; therefore, they were suitable for inclusion in the subsequent regression analysis.

**Table 5:** Results of multicollinearity tests.

Variables	Regression model tolerance	VIF
Age (year)	0.892	1.122
Marital status	0.932	1.073
Household registration	0.62	1.613
Monthly personal income (CNY)	0.816	1.225
Medical expense payment	0.698	1.433
Religion	0.508	1.97
Living arrangement	0.494	2.023
Physical exercise	0.398	2.514
Number of medications	0.357	2.798
Duration of migration	0.621	1.61
Scope of migration	0.41	2.441
Reason for migration	0.355	2.813
Intention to stay	0.879	1.137

We subsequently used multinomial logistic regression to examine the association between two-way social support and health among migrant older adults. Self-rated health was treated as the dependent variable, and the latent classes of two-way social support were included as the independent variable. In the unadjusted model (Model 1), when the balanced social support class (C4) was used as the reference group and “healthy” status was used as the reference category, the odds of reporting “fair health” were 12.667 times greater in the lack of two-way social support class (C1) (OR = 13.667, 95% CI: 4.452–41.949), 10.389 times greater in the mildly support-providing class (C2) (OR = 11.389, 95% CI: 4.322–30.011), and 2.679 times greater in the support-providing dominant class (C3) (OR = 3.679, 95% CI: 1.460–9.274). When comparing “unhealthy” with “healthy” status, participants in the C1 group had 27.576 times higher odds (OR = 28.576, 95% CI: 7.226–113.011), and those in the C2 group had 4.467 times higher odds (OR = 5.467, 95% CI: 1.417–21.086), as shown in Table 6.

**Table 6:** Multivariable logistic regression of two-way social support and health among migrant older adults.

Group	β	S. E.	Wald	OR(95%CI)	*p*
Model 1					
Fair health *VS* healthy					
C1	2.615	0.572	20.885	13.667 (4.452, 41.949)	<0.001
C2	2.433	0.494	24.214	11.389 (4.322, 30.011)	<0.001
C3	1.303	0.472	7.630	3.679 (1.460, 9.274)	0.006
C4 (Reference)	–	–	–	–	–
Model 1					
Unhealthy *VS* Healthy					
C1	3.353	0.702	22.840	28.576 (7.226, 113.011)	<0.001
C2	1.699	0.689	6.082	5.467 (1.417, 21.086)	0.014
C3	1.149	0.645	3.169	3.154 (0.890, 11.170)	0.075
C4 (Reference)	–	–	–	–	–
Model 2					
Fair health *VS* Healthy					
C1	2.457	0.657	13.976	11.668 (3.218, 42.304)	<0.001
C2	2.754	0.545	25.545	15.702 (5.397, 45.682)	<0.001
C3	1.513	0.592	6.527	4.540 (1.422, 14.491)	0.011
C4 (Reference)	–	–	–	–	–
Model 2					
Unhealthy *VS* Healthy					
C1	3.195	0.827	14.930	24.406 (4.827, 123.390)	<0.001
C2	2.129	0.738	8.328	8.404 (1.980, 35.676)	0.004
C3	1.419	0.799	3.158	4.134 (0.864, 19.774)	0.076
C4 (Reference)	–	–	–	–	–

Based on the factors that showed statistically significant differences in the bivariate analyses, we adjusted for covariates—including age, marital status, household registration, monthly personal income, medical expense payment, religion, living arrangement, physical exercise, number of medications, duration of migration, scope of migration, reason for migration, and intention to stay (Model 2)—the overall trend remained consistent. Compared with the C4 group, the odds of reporting “fair health” were increased by 10.668 times in the C1 group (OR = 11.668, 95% CI: 3.218–42.304), 14.702 times in the C2 group (OR = 15.702, 95% CI: 5.397–45.682), and 3.540 times in the C3 group (OR = 4.540, 95% CI: 1.422–14.491). For the comparison between “unhealthy” and “healthy” status, participants in the C1 group had 23.406 times higher odds (OR = 24.406, 95% CI: 4.827–123.390), and those in the C2 group had 7.404 times higher odds (OR = 8.404, 95% CI: 1.980–35.676) (see Table 5).

## 4 Discussion

Our study yielded two primary findings. First, two-way social support among migrant older adults can be classified into four categories, the “lack of two-way social support” class (C1), the “mild support–providing tendency” class (C2), the “support-providing dominant” class (C3), and the “balanced two-way social support” class (C4). The distribution of these categories illustrated a significant imbalance in two-way social support among the migrant older adults, with the balanced two-way social support class (C4) constituting just 14.0% of the sample, while the lack of two-way social support class (C1) (15.6%), the mild support–providing tendency class (C2) (25.6%), and the support-providing dominant class (C3) (44.8%) collectively accounted for the vast majority (86.0%). This finding indicates that a majority of migrant older adults experienced an imbalance between the support they received and the support they provided. Second, it was found that the latent profile of two-way social support was significantly associated with self-rated health among the migrant older adults. The lack of two-way social support (C1) class may indicate the highest health risk, followed by the mild support-providing tendency class (C2) and the support-providing dominant class (C3), while the balanced two-way social support class (C4) may suggest the most favourable health status.

In this study, the risk of self-assessed “unhealthy” status was 23.406 times and 27.576 times greater for individuals in the lack of two-way social support class (C1) than for those in the balanced two-way social support class (C4) when the covariates were controlled for or not (OR = 24.406; OR = 28.576). These findings highlight the significant association between inadequate two-way social support and poorer self-rated health among migrant older adults. The observed vulnerability among members of the lack of two-way social support class (C1) may arise from a confluence of economic, social, and behavioral factors: paying medical expenses out of pocket (92.9%), solitary living rate (87.5%), and engaging in short-term migration (73.2% for less than 1 year). These factors may contribute to difficulties in reconstructing social networks and limited access to health resources. This finding aligns with recent empirical investigations regarding the health risks associated with social isolation and economic pressure among older adults (31–33). The literature indicates that a deficit in social support not only diminishes individuals’ ability to cope with psychosocial stress but also may exacerbate health inequalities through constrained economic resources, increased health care expenditure, and weakened social relationships (34). Overall, insufficient social support may compromise older adults’ psychological well-being and economic stability, thereby increasing their vulnerability during migration and social adaptation.

The health risks observed in the mild support-providing tendency class (C2) and the support-providing dominant class (C3) were lower than those in the lack of two-way social support class (C1), and higher than those in the balanced two-way social support class (C4), indicating the different configurations of social resources are likely to influence health outcomes to varying extents within this demographic. The mild support-providing tendency class (C2) exhibited a tendency towards limited support provision; although their economic conditions were relatively favourable, their financial resources were primarily allocated to familial expenditures and intergenerational support, leading to constraints on health investments and opportunities for external support. This situation indicates that even among more affluent groups, the internal redistribution of resources within families can become a limiting factor in health maintenance. Studies have demonstrated that when economic resources are predominantly directed towards family and intergenerational support, individuals’ investments in health care, physical activity, and regular health screenings may be significantly curtailed (35). The support-providing dominant class (C3), while demonstrating noteworthy social engagement and a sense of responsibility, concentrated their intergenerational responsibilities on caregiving for grandchildren (67.7% migrated for this purpose), leading to a singular form of social support and fragmented health management. In the Chinese social context, which is influenced by traditional family values and a culture of intergenerational support, grandparents tend to prioritize the needs of their adult children and their families, particularly by assuming primary responsibility for grandchild care (9, 36). In this process, their social role is characterized mainly by providing rather than receiving support, resulting in relatively limited attention to their own needs and health management (37). Prior research suggests that undertaking high-intensity intergenerational caregiving roles can result in excessive consumption of both time and psychological resources, adversely impacting the sustainability of personal health management (38, 39). Overall, an imbalance in resource allocation and the concentration of intergenerational responsibilities can undermine individuals’ ability to maintain health. Existing studies suggest that when support investments related to family and intergenerational responsibilities are centred primarily on financial provision and daily caregiving, the absence of complementary social support and health-related resources may have varied effects on older adults’ health (13, 40, 41). Therefore, strategies aimed at enhancing accessibility and incentive mechanisms for health investments in social policy design should specifically target the mild support-providing tendency class (C2) and the support-providing dominant class (C3), while also refining community-level health support networks to harmonize familial obligations with individual health resources (42).

The balanced two-way social support class (C4) exhibited superior health status, likely attributable to the synergistic interplay of balance and mobility. Approximately 88% of individuals in this group cohabited with their children, creating a reciprocal flow of caregiving and emotional support. Existing research has indicated that intergenerational cohabitation and the bidirectional exchange of emotional support contribute to a reduction in loneliness and depression symptoms among older adults while also fostering the continuity of healthy behaviours (43, 44). This integrated form of support, combining emotional support with practical care, constitutes an important social foundation for healthy aging. From the perspective of migration, the majority of short-term migrations (1–3 years, 88%) and migrations targeting elder care (90.0%) have enhanced alignment between living arrangements and health care resources. Previous studies have shown that mobility may promote health by enabling older adults to adjust their place of residence according to their needs, thereby improving the alignment among healthcare services, social resources, and emotional support (45, 46). This “health-motivated mobility” manifests as a significant aspect of active ageing, particularly evident within China’s context of uneven urban and rural health care resource distribution. Moreover, in this class (C4), the health insurance coverage rate reached 62.0%, which was markedly higher than that of the other groups (e.g., only 7.1% in C1). Previous studies have shown that participation in health insurance systems can enhance individuals’ perceived health and health care utilization and, to some extent, alleviate the financial burden of medical expenses (47–49). Consequently, health insurance serves as a key factor in converting the resource advantages associated with migration into tangible health benefits (50, 51). In general, the health advantages enjoyed by the balanced two-way social support group (C4) reflect a dual mechanism of social integration and resource optimization: on the one hand, intergenerational cohabitation establishes a network of emotional and practical caregiving support that enhances psychological and social integration; on the other hand, short-term mobility, coupled with increased health insurance coverage, optimizes access to health care and mitigates economic burdens. Together, the equilibrium of this social support structure and the adaptability of migration strategies represent the critical conditions for maintaining the health of migrant older adults.

In summary, special attention should be given to the health vulnerability of C1. Targeted measures should address issues related to rural household registration constraints, social isolation, and high medical expenses. Policy interventions could include improving cross-regional health insurance portability, enhancing the accessibility of primary health care services, and establishing community-based mutual aid networks to strengthen social connectedness (52–54). Moreover, efforts should be made to promote the transition of the C2 and C3 groups from “one-way contribution” to “mutual support and co-construction.” This transformation can be achieved through integrated policy coordination (e.g., unified health insurance systems), community engagement initiatives (e.g., volunteer service point exchanges for medical benefits), and the creation of family health funds to dynamically balance family responsibilities and individual health needs (55, 56). Future research should further explore the long-term health effects of two-way social support and the mechanisms of transitions between different support types, thereby providing empirical evidence and policy guidance to promote health equity among older adults.

## 5 Limitations

This study has some limitations. First, convenience sampling was used, and participants were recruited exclusively from Huzhou, Zhejiang Province, China. This may have introduced selection bias and limited the representativeness and generalizability of the findings to migrant older adults living in rural and other areas. Second, health status was assessed primarily using self-rated health and chronic disease status, which may not fully capture the overall health of older adults. Third, because this study employed a cross-sectional design, the findings can only demonstrate associations between latent profiles of two-way social support and health status, and causal relationships cannot be inferred. Finally, several potential confounding variables that may influence both social support and health outcomes, such as depression and cognitive impairment, were not included in the analysis. Their omission may have introduced residual confounding and partly explained the observed associations. Future studies should adopt longitudinal and multicenter designs involving more diverse regions, incorporate more comprehensive, objective, and functional health indicators, adequately account for potential confounding variables, and further validate the associations between two-way social support profiles and health outcomes.

## 6 Conclusion

In this study, latent profile analysis (LPA) identified four distinct profiles of two-way social support among migrant older adults in China: the “lack of two-way social support” class, the “mild support-providing tendency” class, the “support-providing dominant” class, and the “balanced two-way social support” class. These profiles were associated with health outcomes. Specifically, individuals in the “lack of two-way social support” class faced the greatest health risk, whereas those in the “balanced two-way social support” class presented the most favourable health status. These findings indicate that different profiles of two-way social support are associated with differences in health status among migrant older adults. The results may help identify subgroups characterised by relatively limited two-way social support and less favourable health status. These results provide empirical evidence for the development of targeted interventions. They may also provide a reference for future efforts to strengthen social support networks, improve access to medical and social resources, and promote social integration, thereby addressing the social support and health-related needs of migrant older adults.

## Data Availability

The raw data supporting the conclusions of this article will be made available by the authors, without undue reservation.
